# Systemic inflammatory markers in patients with polyneuropathies

**DOI:** 10.3389/fimmu.2023.1067714

**Published:** 2023-02-13

**Authors:** Patricia García-Fernández, Klemens Höfflin, Antonia Rausch, Katharina Strommer, Astrid Neumann, Nadine Cebulla, Ann-Kristin Reinhold, Heike Rittner, Nurcan Üçeyler, Claudia Sommer

**Affiliations:** ^1^Department of Neurology, University Hospital of Würzburg, Würzburg, Germany; ^2^Department of Bioanalytics, Bionorica research GmbH, Innsbruck, Austria; ^3^Department of Anesthesiology, University Hospital of Würzburg, Würzburg, Germany

**Keywords:** cytokines, polyneuropathy, cerebrospinal fluid, neurofilament light chain, blood CSF barrier

## Abstract

**Introduction:**

In patients with peripheral neuropathies (PNP), neuropathic pain is present in 50% of the cases, independent of the etiology. The pathophysiology of pain is poorly understood, and inflammatory processes have been found to be involved in neuro-degeneration, -regeneration and pain. While previous studies have found a local upregulation of inflammatory mediators in patients with PNP, there is a high variability described in the cytokines present systemically in sera and cerebrospinal fluid (CSF). We hypothesized that the development of PNP and neuropathic pain is associated with enhanced systemic inflammation.

**Methods:**

To test our hypothesis, we performed a comprehensive analysis of the protein, lipid and gene expression of different pro- and anti-inflammatory markers in blood and CSF from patients with PNP and controls.

**Results:**

While we found differences between PNP and controls in specific cytokines or lipids, such as CCL2 or oleoylcarnitine, PNP patients and controls did not present major differences in systemic inflammatory markers in general. IL-10 and CCL2 levels were related to measures of axonal damage and neuropathic pain. Lastly, we describe a strong interaction between inflammation and neurodegeneration at the nerve roots in a specific subgroup of PNP patients with blood-CSF barrier dysfunction.

**Conclusion:**

In patients with PNP systemic inflammatory, markers in blood or CSF do not differ from controls in general, but specific cytokines or lipids do. Our findings further highlight the importance of CSF analysis in patients with peripheral neuropathies.

## Introduction

Polyneuropathy (PNP) is a term to describe a group of diseases with peripheral nerve dysfunction of various etiologies. Symptoms may affect the motor, sensory and/or autonomic system, and in 50% of the cases, patients experience neuropathic pain. Why some patients with PNP have pain and others not, is unknown, and even PNPs with the same etiology may be painful or painless (1). The available treatments have modest efficacy in reducing pain and present considerable side effects (2, 3). One approach toward better symptom control and potentially causative treatment might be to find common pathophysiologic pathways in PNP of different etiologies.

Although merely 14%-20% of PNP have a definite immune-related cause (4), inflammatory processes have been found to be involved in neuro-degeneration, -regeneration and pain in neuropathies of different origin (5–7). One of the main pro-inflammatory pathways that are activated upon damage is toll-like receptor (TLR) 4 mediated. Stimulation of TLR4 results in the activation of the nuclear factor k B (NFkB) pathway and the release of inflammatory cytokines, including tumor necrosis factor-alpha (TNF-α), interleukin (IL)-1β, IL-6, IL-8, IL-10, or chemokines such as the CC-chemokine ligand (CCL) 2 (8). This pathway can in turn be regulated by other mediators. For instance, the NAD-dependent deacetylase sirtuin 1 (SIRT1) has been suggested to be able to inhibit the pro-inflammatory cascade by deacetylating the p65 subunit of NFkB (9). Fractalkine (Cx3CL1) is an algesic chemokine which is cleaved from neurons and activates glial cells (10). Furthermore, small fragments of RNA, or microRNAs (miR), can regulate the expression of genes coding for pro- and anti-inflammatory proteins and have been found altered in different neuropathies of the central or peripheral nervous system, nociception and pain (11). While many miRs might be involved in the development of neuropathic pain, miR-146a-5p, miR-132-3p and miR-155-5p participate in the regulation of the NFkB pathway and have been described altered in patients with painful PNP (12–14). In addition, alterations in ion channels such as voltage gated sodium channels or those of the transient receptor potential cation channel subfamily like TRPV1 might lead to enhanced excitatory responses in nociceptors and promote axonal degeneration and the release of pro-inflammatory mediators, exacerbating the immune response (15). Furthermore, TRPV1 is expressed in blood mononuclear cells, and a direct role in cytokine production and immune responses has been proposed (16, 17).

Several lipid compounds, such as prostaglandins (PGs) or thromboxanes, are known to be involved in inflammation and pain, and can in turn be upregulated upon inflammation or tissue injury. Moreover, high levels of PGs have been described in serum from patients with peripheral neuropathies, as well as in the spinal cord of animal models with peripheral injury (18, 19). In fact, non-steroidal anti-inflammatory drugs (NSAIDs), a very common treatment against pain, have anti-inflammatory and analgesic properties mediated by blocking the synthesis of PGs and thromboxanes (18, 20, 21). Short-chain and long-chain acylcarnitines can be found upstream their synthesis pathway and have been reported upregulated in sera, nerve and spinal cord in animal models of neurodegeneration, in particular, palmitoylcarnitine (C16:0) and oleoylcarnitine (C18:1) (20, 22–24).

In PNP, signs and symptoms are typically length-dependent and thus more severe in the distal extremities of the body, such as feet and hands, than in the proximal regions or in the torso. Previous studies found a local upregulation of inflammatory mediators such as IL-2, IL-6, IL-8 or IL-10 in skin or nerve from patients with PNP (5, 25). The study of local inflammation, especially in the nerve, is difficult in humans, since it implies an invasive nerve biopsy, while systemic samples, such as blood and cerebrospinal fluid (CSF), are drawn and analyzed routinely. Since several inflammatory cytokines have been seen upregulated locally in patients with PNP, we studied whether some of these markers might be found systemically in sera or CSF. Different studies have shown a high variability in the cytokines present in serum from patients with PNP or nerve root compression, associated with the severity and symptoms of the neuropathy (26–31). Furthermore, extensive studies have shown that the CSF is directly altered by diseases of the central nervous system, while in diseases of the peripheral nervous system (PNS), differences in the levels of cytokines in CSF have yet to be explained. A factor that can influence these levels is the integrity of the barrier between blood and CSF, also called blood-CSF-barrier (B-CSF barrier) (32–34) or blood brain barrier (BBB) (35).

We hypothesized that PNP is associated with enhanced systemic inflammation that may correlate with the severity of the disease and the development of neuropathic pain. To test our hypothesis, we performed a comprehensive analysis of the protein, lipid, and gene expression of selected pro- and anti-inflammatory markers in blood and CSF from patients with PNP and controls. Furthermore, we measured the levels of neurofilament light-chain (NFL), as a cytoskeletal protein expressed by neurons and released upon cell damage, to assess the level of neurodegeneration present in patients with PNP (36), and to correlate NFL levels with the degree of inflammation. We also aimed to identify new systemic markers that might be important in the pursuit of a more accurate diagnosis and the prediction of neuropathic pain.

## Materials and methods

### Patient recruitment and diagnostic assessment

Between 2019 and 2021, 28 patients with PNP were prospectively recruited at the Department of Neurology, University of Würzburg, Germany, where they were seen for diagnostic work-up. In addition, 10 patients with acute headaches of unclear etiology were used as disease controls for the CSF analysis, and serum data from twelve healthy volunteers were included in the analysis to determine the inflammatory state of the headache group. Our study was approved by the Würzburg Medical Faculty Ethics Committee (# 15/19 and # 242/17) and all participants gave written informed consent prior to inclusion.

Diagnoses were based on history taking and neurological examination, laboratory studies, and nerve conduction examinations. All patients underwent laboratory tests including full blood count, electrolytes, kidney and liver function tests, C-reactive protein, thyroid stimulating hormone, vitamin B12, HbA1c, oral glucose tolerance test (OGTT), screening for autoimmune antibodies (i.e., ANA, ENA, ANCA, anti-ganglioside antibodies), and lumbar puncture. Electrophysiological assessment with nerve conduction studies of the affected nerves including tibial motor nerve and sural sensory nerve was performed in all PNP patients.

To rate the severity of the neuropathy, we used standardized scales, including the modified Toronto clinical neuropathy score (mTCNS), the overall disability sum score (ODSS) and the Medical Research Council-sumscore (MRC-sumscore). Depression was assessed with the “Allgemeine Depressionsskala” (ADS) (37). Pain was evaluated by a numerical rating scale (NRS) from 0 (no pain) to 10 (worst pain), the neuropathic pain symptom inventory (NPSI) and the graded chronic pain scale (GCPS). After the diagnostic work-up, the examining neurologists categorized the neuropathies into a mild (1), moderate (2) or severe (3) clinical phenotype.

### Sample collection

From each patient, venous blood was drawn in the morning between 8 a.m. and 9 a.m. into S-Monovette^®^ Serum-Gel Tubes (Sarstedt, Nümbrecht, Germany) and Tempus™ Blood RNA Tubes (Thermo Fisher Scientific, Waltham, MA, USA). For the collection of sera, whole blood was left to clot for 30 min at room temperature and later centrifuged for 10 min at 1200 g. Supernatant was aliquoted and stored at -20°C until further analysis. Tempus™ Blood RNA Tubes were immediately shaken for 30 s to lyse the cells and stabilize the RNA and stored at -80°C until extraction.

CSF samples were obtained after a lumbar puncture at the L4/5 level. The amount of CSF taken varied between 10-15 ml. Samples of 2 ml were aliquoted and subsequently stored at -20 °C.

### Gene expression analysis

RNA was extracted from Tempus™ Blood RNA Tubes following the manufacturer’s protocol (38, 39) from the MagMAX™ for Stabilized Blood Tubes RNA Isolation Kit (Thermo Fisher Scientific, Waltham, MA, USA). RNA quality and quantity were assessed with a NanoDrop™ One (Thermo Fisher Scientific, Waltham, MA, USA), and RNA was stored at -80°C.

For cDNA synthesis from mRNA, TaqMan Reverse Transcription reagents (Thermo Fisher Scientific, Waltham, MA, USA) were used. 250 ng mRNA of each sample were pre-incubated with 5 µl random hexamer at 85°C for 3 min. Next, 10 μL 10× PCR buffer, 22 μL MgCl_2,_ 20 μL deoxyribonucleoside triphosphate, 6.25 μL multiscribe reverse transcriptase and 2 μL RNase inhibitor were added per sample. Lastly, reaction was performed under these conditions: annealing (25°C, 10 min), reverse transcription (48°C, 60 min), and enzyme inactivation (95°C, 5 min).

For miRNA, reverse transcription was carried out with the miRCURY LNA RT Kit (Qiagen, Hilden, Germany). 10 ng of RNA were mixed with 2 μL of 5x reaction buffer, 5 μL of nuclease free water and 1 μL of enzyme mix, per sample. Reaction was performed using the following program: reverse transcription (42°C, 60 min) and enzyme deactivation (95°C, 5 min).

Reactions were carried out on a PRISM 7700 Cycler (Applied Biosystems, Waltham, MA, USA) and transcribed cDNA was stored at -20°C until further analysis.

Real time qPCR of mRNA and miRNA targets was performed to analyze gene expression using the StepOnePlus Real-Time PCR System (Thermo Fisher Scientific, Waltham, MA, USA). For mRNA, RT-qPCR was carried out with TaqMan qRT-PCR reagents (all Thermo Fisher Scientific, Waltham, MA, USA) and pre-designed assays. For target normalization, different endogenous controls were used: ribosomal protein L13a (RPL13A), actin beta (ACTb), Hydroxymethylbilane Synthase (HMBS) and TATA-Box binding protein (TBP) were tested. TBP was the most stable across groups, and thus selected as suitable endogenous control. For each reaction, 3.5 µl cDNA (8.75 ng cDNA) was mixed with 0.5 µl nuclease free water, 5 µl Fast Advanced Mastermix and 0.5 µl TBP primer and 0.5 µl target primer (see list of primers in **Table 1**).

**Table 1:** List of primer assays.

Taqman Primer*	Assay Number
TRPV1	Hs00218912_m1
TLR4	Hs00152939_m1
SIRT1	Hs01009006_m1
TNFα	Hs00174128_m1
IL-1β	Hs00174097_m1
IL-6	Hs00174131_m1
IL-8	Hs00174103_m1
CCL2	Hs00234140_m1
IL-10	Hs00174086_m1
TBP	Hs00427620_m1
SYBR Green Primer^#^	Assay Number
hsa-miR-132-3p	YP00206035
hsa-miR-146a-5p	YP00204688
hsa-miR-155-5p	YP00204308
hsa-miR-16-5p	YP00205702
5s rRNA	YP00203906

*Taqman Primers were purchased from Thermo Fisher Scientific, Waltham, MA, USA.

#SYBR Green Primers were purchased from Qiagen, Hilden, Germany.

TRPV1, transient receptor potential cation channel subfamily V member 1; TLR4, toll-like receptor 4; SIRT1, sirtuin 1; TNFα, tumor necrosis factor α; IL, interleukin; CCL2, CC-chemokine ligand 2; TBP, TATA-Box binding protein; miR, microRNA, rRNA, ribosomalRNA.

For miRNA, the miRCURY LNA SYBR Green PCR Kit (Qiagen, Hilden, Germany) and pre-designed miRCURY LNA miR PCR assays (Qiagen, Hilden, Germany) were used. Based on previous experience from our group (12, 13) and on the recommendations by TaqMan Advanced miRNA Assays (https://assets.thermofisher.com/TFS-Assets/GSD/Reference-Materials/identifying-mirna-normalizers-white-paper.pdf), the ribosomal RNA 5s and hsa-miR-16 were tested as endogenous controls. Due to differences found in the expression of 5s between groups, hsa-miR-16 was selected as suitable endogenous control, based on its comparability and standard deviation across groups and samples. Each miRNA was run adding 5 µl 2× miRCURY SYBR Green Master Mix with 1 µl ROX per 50 µl and 1 µl primer (see list of primers in **Table 1**) to 4 µl of 1:80 diluted cDNA.

Each mRNA and miRNA was amplified in triplicates and relative quantitation (RQ) values were obtained by the StepOnePlus™ Software v2.3 (Thermo Fisher Scientific, Waltham, MA, USA) using interplate calibrators through the 2-ΔΔCt method.

### Protein analysis

Cytokine levels were measured in serum and CSF using the Ella™ technology (ProteinSimple, San Jose, Cal, USA). Ella™ is a fully automated cartridge-based system that allows you to perform multiple sample, multi-analyte immunoassays with the specificity of a traditional single-plex ELISA (enzyme-linked immunosorbent assay). Samples were thawed on ice and more than two freeze-thaw cycles were avoided. Samples were diluted 1:1 in the appropriate sample diluent from each kit and 50 µl were added to each sample well of the Simple Plex™ cartridge, after 1 ml of washing buffer was added to their corresponding wells. Each sample was measured in triplicates, and the levels of each cytokine were displayed in pg/ml.

### Lipid analysis

Serum and CSF samples were quantitatively analyzed for their levels of the endogenous metabolites carnitine (CAR), palmitoylcarnitine (PC) and oleoylcarnitine (OC) by LC-MS/MS (liquid chromatography coupled with tandem mass spectrometry).

All samples were thawed on ice for the first time for this analysis, in order to avoid freeze-thaw cycles, and processed within two years upon collection. Serum samples were analyzed at a neat dilution and after applying a 1:10 dilution step in 70% (v/v) ethanol. For sample preparation, 50 µl of serum or CSF samples were mixed with 150 µl internal standard solution (100 ng/ml acetylcarnitine-d3 in acetonitrile). Samples were centrifuged (13000 rpm, 1.5 min) and the supernatant was used for analysis.

Chromatographic separation was performed with a UHPLC System (1290 Infinity series, Agilent) using a Waters Acquity UHPL CSH™ Fluoro-Phenyl (75 × 2.1 mm, 1.7 µm) column. The injection volume was 2 µl and the flow rate was set to 0.3 mL/min. For the chromatographic separation a gradient was run using 0.1% formic acid in water (solvent A) and 0.1% formic acid in acetonitrile (solvent B). Detection was performed by means of a triple quadrupole mass spectrometer (API 4000^®^, Sciex) in multiple reaction monitoring (MRM) mode. Measurements were carried out in the positive electrospray ionization (ESI) mode. The LC-MS/MS system was operated with the Software Analyst^®^, Version 1.7.1 (Sciex).

Calibration samples were prepared in 30% acetonitrile and covered calibration ranges of 5 - 500 ng/ml for PC and OC and 5 - 1500 ng/ml for CAR. To verify method performance at medium analyte concentrations, QC samples with a concentration of 50.00 ng/ml CAR, OC and PC were prepared in 30% acetonitrile or in a surrogate matrix comparable to the analyzed biological samples (2% bovine serum albumin (BSA) or PBS buffer pH 7.4 diluted 1:10 in 30% acetonitrile were used to mimic serum or CSF, respectively).

Correlation coefficients of 0.9996 (CAR, OC) and 0.9992 (PC) were obtained. Accuracies of the calibration samples were found within +/-15% or within +/-20% for the lowest calibration level, respectively. Mean accuracies for all QC samples were found between 87.07% and 109.77% with CVs < 15%, indicating an acceptable method performance for all matrices investigated.

Further analytical information can be found in the **Supplementary Material**.

### Statistical analysis and visualization

Statistical analysis was performed in SPSS 27 (IBM, Armonk, NY, USA), where the Shapiro-Wilk test was used to determine the normal distribution of the data. For parametric data, a T-test was used for comparison between two groups and a Pearson test was performed for correlations. In non-parametric data, the Mann–Whitney U Test was applied for comparison of two groups, and the Spearman test was used for correlations. Data results were plotted in GraphPad Prism 9 (GraphPad Software, Inc., La Jolla, CA, USA) for visualization. Graphical images were incorporated from Smart Servier Medical Art, https://smart.servier.com/, under the Creative common Attribution 3.0 Unported Licence.

## Results

### Clinical characteristics of patient cohort

Baseline characteristics of the study group and the diagnostic subgroups are summarized in **Table 2**. Patients were included if they presented for diagnostic work-up for their PNP, including lumbar puncture. To be included, they further needed to have either no pain (NRS = 0) or pain ≥ 4 at the time of admission. Patients with an NRS between one and three were excluded from the cohort. After applying the exclusion criteria, twenty-eight patients with PNP of different etiologies were included [median body max index (BMI) 28.3, range 19.2-35.1; median age 54.5 years, range 20–80]. The median disease duration was 2.5 years (range 0.02–29 years). Nine patients were diagnosed with an inflammatory neuropathy including non-systemic vasculitis (six patients), chronic inflammatory demyelinating polyneuropathy (CIDP) (two patients) and multifocal motor neuropathy (MMN) (one patient). Nineteen patients were classified as non-inflammatory, including idiopathic neuropathy (nine patients), hereditary neuropathy (five patients), a neuropathy caused by vitamin B deficiency (three patients) or diabetic neuropathy (two patients).

**Table 2:** Description and diagnostic subgroups of patient cohort.

Item	Number (% of entire group)
**M, F (*N*)**	22, 6
**Median BMI (range)**	28.3 (19.2-35.1)
**Median age (range)**	54.5 years (20-80)
**Median disease duration (range in years)**	2.5 years (0.02-29)
Diagnostic subgroups (*N* and % of entire group):	
**Idiopathic neuropathy**	9 (32.1%)
**Vasculitic neuropathy**	6 (21.4%)
**Hereditary neuropathy**	5 (17.9%)
**Vit B1/B6/B9/B12 deficiency**	3 (10.7%)
**Diabetic neuropathy**	2 (7.1%)
**CIDP**	2 (7.1%)
**Multifocal motor neuropathy (MMN)**	1 (3.6%)
Analysis subgroups (*N*):	
**Painless, painful**	14, 14
**Inflammatory, non-inflammatory neuropathy**	9, 19
Patients with treatment, without treatment (*N*)*:	15, 13
**Immunosupressory/immunomodulatory treatment** (Corticoids, immunoglobulins, NSAIDs)	6
**Pain treatment^#^**	12
Anti-neuropathic analgesics	11
Opioids	4

Numbers add up to >28 (*) and >12 (#) because some patients had more than one treatment.

Patients were classified as painless when they presented an NRS = 0 (fourteen patients; 50%), and as painful with an NRS ≥ 4 (fourteen patients; 50%). From the full cohort of PNP patients, at the time of inclusion fifteen patients had been treated for their neuropathy with either an immunosuppressive/immunomodulatory drug (six patients) and/or pain treatment (twelve patients), while thirteen received no treatment.

Further laboratory and electrophysiological data are given in **Table 3**. The median CRP value in serum was 0.24 mg/dl (range 0-2.37 mg/dl). In CSF, patients presented a median of 2 leukocytes/µl (range 0-6 cells/µl) and a median total protein of 46.3 mg/dl (range 19.3-194.6 mg/dl). Nerve conduction studies showed a median sural nerve sensory nerve action potential (SNAP) of 4.9 µV (range 2.5-12.6 µV) with a median nerve conduction velocity (NCV) of 45.9 m/s (range 32.4-57.3 m/s), and in the tibial nerve a median compound motor action potential (CMAP) upon stimulation at the ankle of 5.4 mV (range 0.1-23.4 mV) and a median nerve conduction velocity (NCV) of 38.3 m/s (range 26.8-52.2 m/s).

**Table 3:** Clinical findings in PNP patients versus controls.

Item	Normal range	PNP	AH	UH
N	NA	28	10	5
Serum findings				
**Median CRP (mg/dl) (range)**	≤0.5	0.24 (0-2.37)	0.23 (0-11.39)	0 (0-0.34)
**Median NFL (pg/ml) (range)**	NA	27.35 (8.3-2856)**/**^##^**	10.80 (3.04-48.90)	4.92 (3.04-26.90)
Lumbar puncture				
**Median cells/µl (range)**	≤4	2 (0-6)	0.5 (0-3)	0 (0-3)
** Median CSF protein (mg/dl) (range)**	≤50	46.3 (19.3-194.6)	27.6 (16.9-44)	30.1 (21.2-38.8)
** Median NFL (pg/ml) (range)**	NA	627.0 (155.0-8535)***/**^##^**	291.0 (134.0-489.0)	199.0 (123.0-413.0)
** Median NFL Ratio (CSF/Serum)**	NA	26.14 (1.9-75.9)	28.53 (8.4-66.1)	56.53 (15.35-66.06)
Electrophysiology				
Sural nerve				
**Median SNAP (µV) (range)**	≥5/10 (Age ≥65y/<65y)	4.9 (2.5-12.6)	NA	NA
**Median NCV(m/s) (range)**	≥40	45.9 (32.4-57.3)	NA	NA
Tibial nerve				
**Median Distal CMAP (mV) (range)**	≥10	5.4 (0.1-23.4)	NA	NA
**Median NCV (m/s) (range)**	≥40	38.3 (26.8-52.2)	NA	NA

Patients with polyneuropathies (PNP) were compared to the control group acute headaches (AH) (10), and to the subgroup unclear headaches (UH) (5/10). CRP, C-reactive protein; NFL, neurofilament light chain, CSF, cerebrospinal fluid; SNAP, sensory nerve action potential; NCV, nerve conduction velocity; CMAP, compound motor action potential. Kruskal-Wallis or T-test was performed between PNP and AH (*) or UH (^#^). **/^##^, p < 0.01; ***, p < 0.001.

### Control group

**Figure 1:**
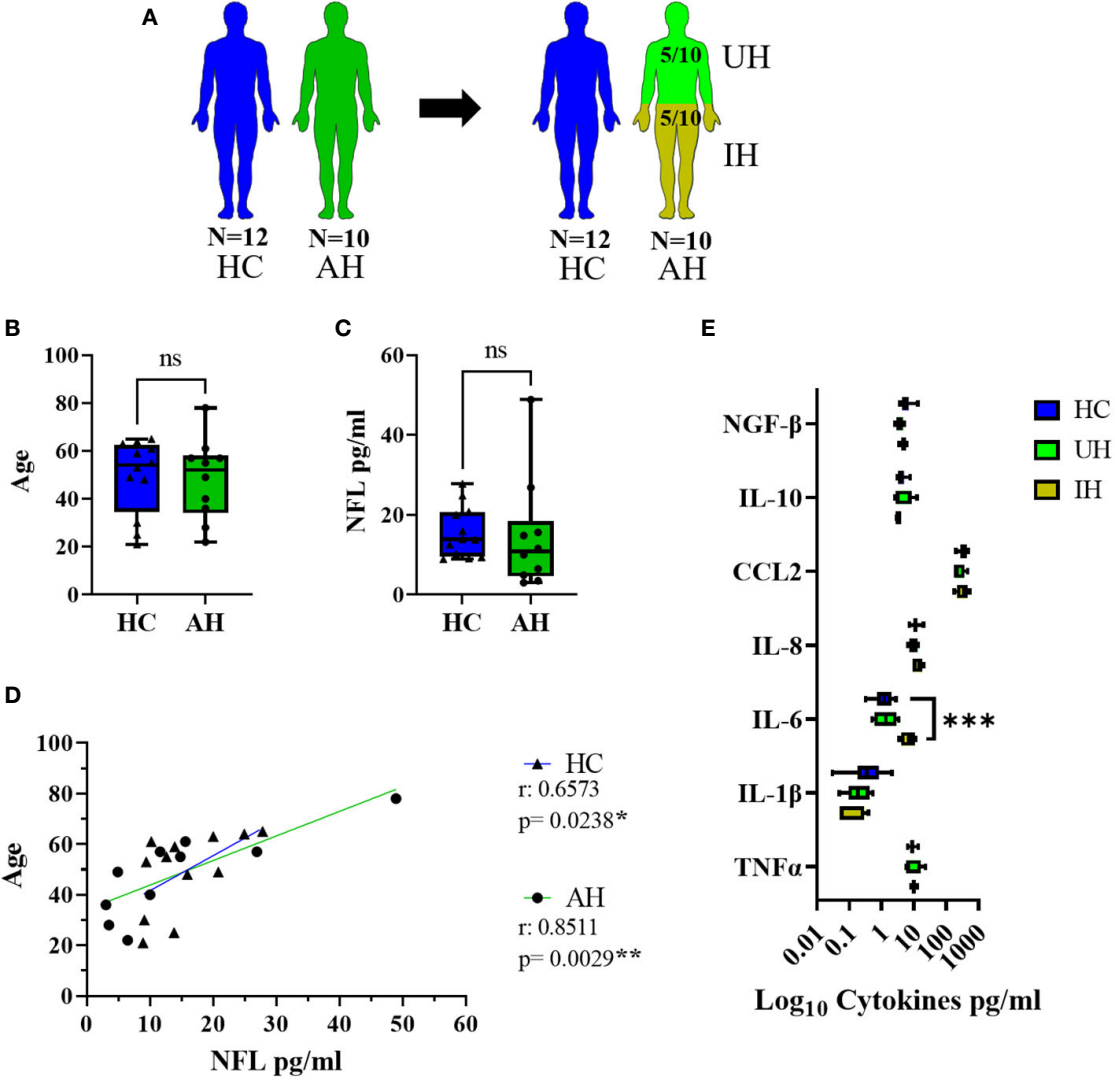
Description of the control groups. **(A)** Visual representation of the healthy controls (HC) (blue) and acute headaches group (AH) (green), further divided into unclear headaches (UH, light green) and inflammatory headaches (IH, olive green). **(B)** Age values in HC and AH. **(C)** NFL levels in serum in pg/ml between HC and AH. **(D)** Correlation between age and NFL levels in serum in pg/ml in HC and AH. **(E)** Log10 representation of cytokine values in serum from HC, UH and IH. ns, not significant; *, p < 0.05; **, p < 0.01; ***, p < 0.001. All graphical images were incorporated from Smart Servier Medical Art, https://smart.servier.com/, under the Creative common Attribution 3.0 Unported Licence.

Patients that presented with acute headaches of unclear etiology (AH) and had a lumbar puncture to exclude meningitis or a subarachnoid bleed were used as controls for the CSF data. Only patients that had less than four leukocytes/µl in their CSF and normal protein values were included. After full clinical work-up, five of these patients had some signs of inflammation (elevated CRP, n = 3; sinusitis, n = 1; or elevated ANA and ANCA titers, n = 1). Thus, the AH group was divided into two groups (unclear headache, UH, and inflammatory headache, IH), and only patients with UH were further used as controls for inflammatory markers (**Table 4**, **Figure 1A**). Twelve healthy volunteers (HC) were included as controls for NFL and inflammatory markers in serum. HC and AH did not differ in age or NFL levels in pg/ml in serum (**Figures 1B, C**), and in both groups a positive correlation between age and NFL was present (HC, p < 0.05; AH, p < 0.01) (**Figure 1D**). When separating AH into UH and IH, NFL levels did not differ between groups, but IL-6 was higher in IH in comparison to HC (p < 0.001) (**Figure 1E**). These results supported our decision to continue the study on inflammatory markers with the UH exclusively, while the whole cohort (AH) was used for the NFL analysis.

**Table 4:** Description of the control groups.

Item	Number (% of entire group)		
	Acute headaches (AH)		Healthy controls (HC)
**M, F (N)**	6, 4		6,6
**Median age (range)**	52 years (22-78)		54 years (21-65)
Diagnostic subgroups (*N* and % of entire group):			
**Unclear headaches (UH)**	5 (50%)		NA
**Infectious/inflammatory headaches (IH)**	5 (50%)		NA
**Median NFL pg/ml in serum (range)**	10.80 (3.04-48.90)		13.85 (8.93-27.80)
	UH	IH	HC
**Median NFL pg/ml in serum (range)**	4.92 (3.04-26.90)	14.80 (10.00-38.90)	13.85 (8.93-27.80)
Median of cytokine levels in serum (range):			
**TNFα pg/ml**	6.78 (6.04-23.20)	9.89 (8.22-12.90)	8.94 (6.51-13.55)
**IL-1β pg/ml**	0.19 (0.05-0.54)	0.06 (0.05-0.4)	0.35 (0.03-2.11)
**IL-6 pg/ml**	1.36 (0.51-3.48)	7.04 (3.52-11.50)***	1.22 (0.33-2.80)
**IL-8 pg/ml**	9.38 (6.72-13.70)	14.40 (9.19-20.60)	11.65 (7.35-19.90)
**CCL2 pg/ml**	198 (190-454)	340 (179-531)	357 (201-470)
**IL-10 pg/ml**	3.06 (2.63-12.50)	3.55 (2.89-3.62)	3.95 (3.07-7.69)
**NGF-β pg/ml**	3.34 (2.66-5.00)	4.77 (3.52-5.99)	5.04 (3.76-13.50)

NFL, neurofilament light-chain; TNFα, tumor necrosis factor α; IL, interleukin; CCL2, CC-chemokine ligand2, NGF-β, nerve growth factor β. (*) Kruskal-Wallis test was performed between HC and UH or IH.***, p < 0.001.

### NFL levels in serum and CSF from PNP patients

**Figure 2:**
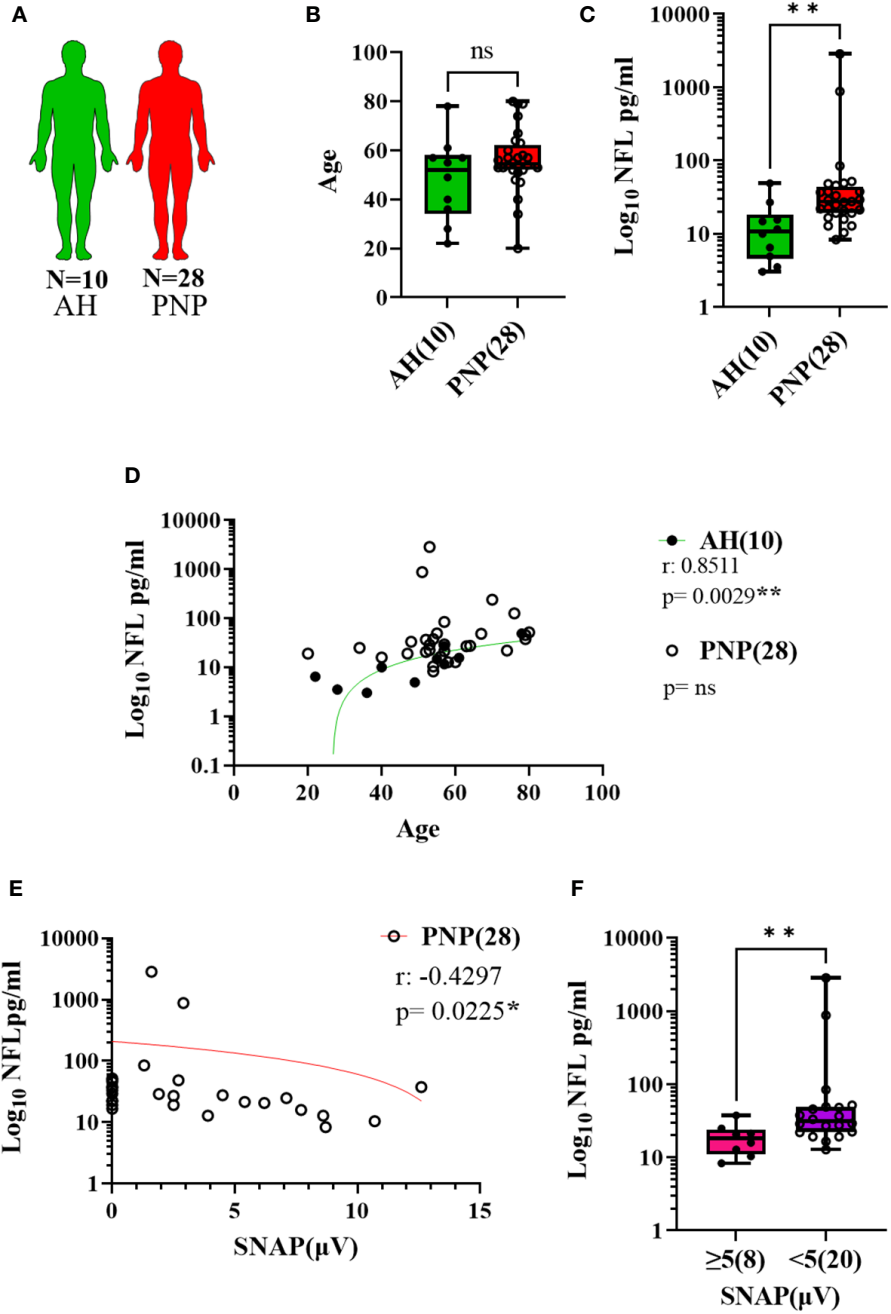
NFL levels in serum from patients with PNP. **(A)** Visual representation of the analyzed 10 AH (green) and 28 PNP patients (red). **(B)** Age values in AH and PNP. **(C)** Log 10 of NFL levels in pg/ml between AH and PNP. **(D)** Correlation between age and log 10 of NFL levels in pg/ml in AH and PNP. **(E)** Correlation between log10 of NFL levels in pg/ml and SNAP. **(F)** Log10 of NFL levels in pg/ml between PNP patients with normal (≥ 5µV) (pink) and abnormal (< 5µV) (purple) sural nerve SNAP. ns, not significant; *, p < 0.05; **, p < 0.01. All graphical images were incorporated from Smart Servier Medical Art, https://smart.servier.com/, under the Creative common Attribution 3.0 Unported Licence.

NFL was measured in serum and CSF in twenty-eight patients with PNP in comparison to ten age-matched AH (**Figures 2A, B**). PNP patients had higher serum levels of NFL than AH (p < 0.01) (**Figure 2C**). There was a positive correlation between age and NFL levels in AH (p < 0.01) but not in PNP patients (**Figure 2D**). NFL levels correlated negatively to sural nerve SNAP in PNP patients (**Figure 2E**), which translated into higher levels of NFL in patients with abnormal SNAP values (< 5 µV) in comparison to those with normal SNAP (≥ 5 µV) (p < 0.01) (**Figure 2F**).

**Figure 3:**
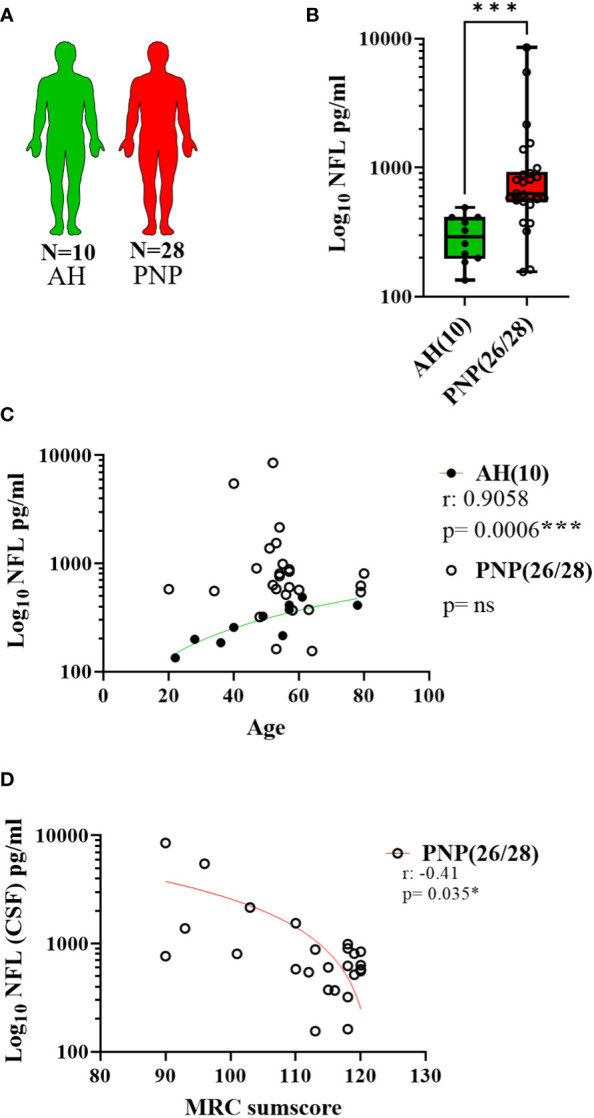
NFL levels in CSF from patients with PNP. **(A)** Visual representation of the analyzed 10 AH (green) and 28 PNP patients (red). **(B)** Log 10 of NFL levels in pg/ml between AH and PNP. **(C)** Correlation between age and log 10 of NFL levels in pg/ml in AH and PNP. **(D)** Correlation between the levels of NFL levels in pg/ml (Log10) and the MRC sumscore in PNP patients. ns, not significant; *, p < 0.05; ***, p < 0.001. All graphical images were incorporated from Smart Servier Medical Art, https://smart.servier.com/, under the Creative common Attribution 3.0 Unported Licence.

The same analysis was performed with CSF samples (**Figure 3A**) and we observed higher levels of NFL in PNP patients in comparison to AH (p < 0.001) (**Figure 3B**). Again, a correlation between age and NFL was present in AH (p < 0.001) but not in PNP patients (**Figure 3C**). Furthermore, our results showed a negative correlation between the levels of NFL in CSF and the MRC sumscore (**Figure 3D**), therefore suggesting an involvement of NFL release in the severity of the neuropathy.

### Gene expression of pro- and anti-inflammatory markers in whole blood

**Figure 4:**
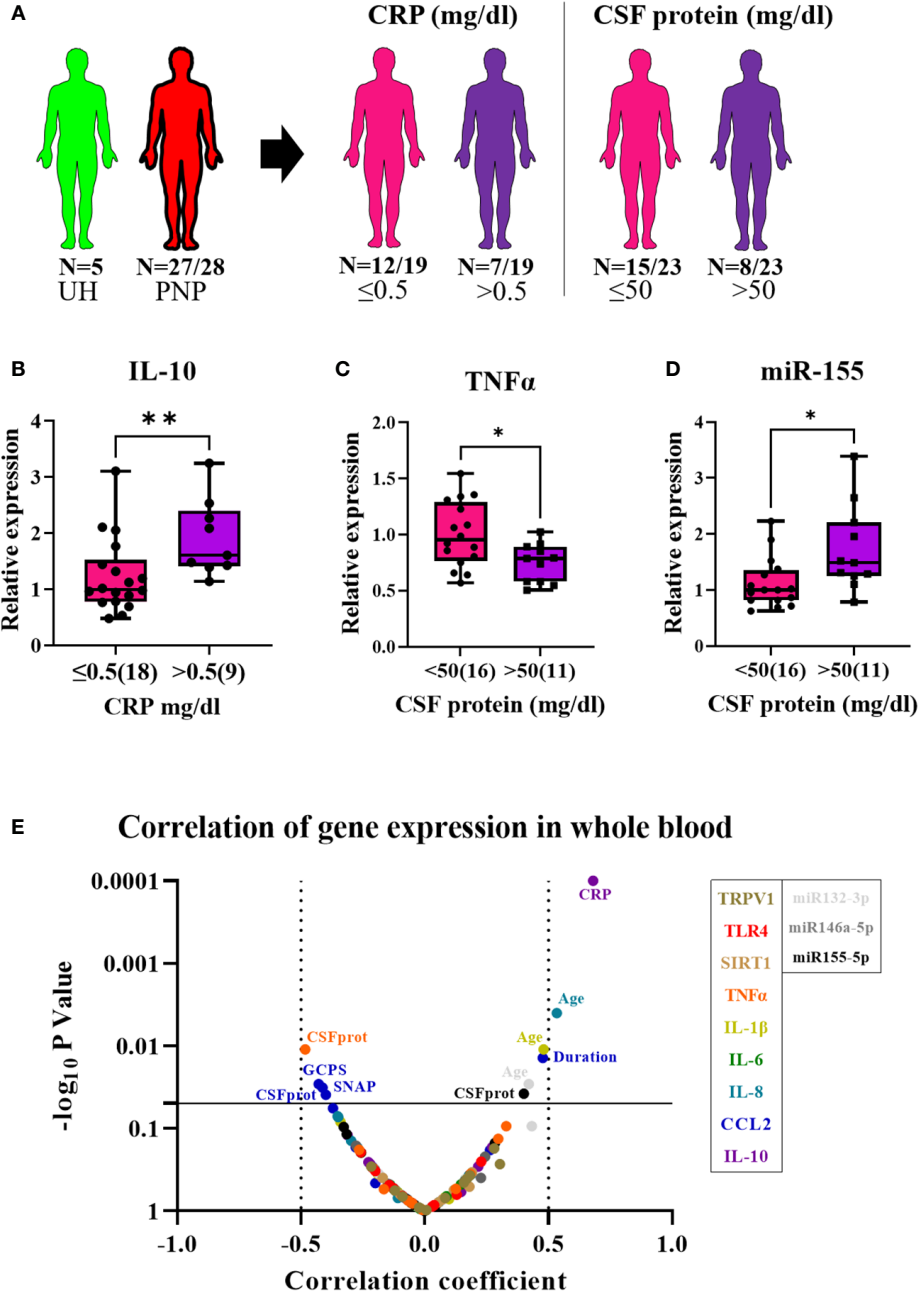
Relative gene expression of pro- and anti-inflammatory markers in whole blood from patients with PNP. **(A)** Visual representation of the analyzed 5 UH (light green) and 27 out of 28 PNP patients (red), further divided into 18 PNP patients with normal (≤0.5mg/dl, pink) and seven with increased (> 0.5 mg/dl, purple) CRP levels in serum, and into 16 PNP patients with normal (≤ 50 mg/dl, pink) and seven with abnormal (> 50 mg/dl, purple) protein levels in CSF. **(B)** Relative expression of IL-10 in PNP patients with increased CRP in comparison to those with normal values. Relative expression of TNFα **(C)** and miR-155 **(D)** in PNP patients with normal and abnormal protein levels in CSF. **(E)** Volcano plot of the Spearman multivariate correlations between each studied target and the neuropathy scores. *, p < 0.05; **, p < 0.01. All graphical images were incorporated from Smart Servier Medical Art, https://smart.servier.com/, under the Creative common Attribution 3.0 Unported Licence.

In twenty-seven out of twenty-eight PNP patients and five UH, we analyzed the gene expression of the receptors TRPV1 and TLR4; the deacetylase SIRT1; the pro-inflammatory cytokines TNFα, IL-1β, IL-6 and IL-8, and chemokine CCL2; the anti-inflammatory cytokine IL-10; and the microRNAs miR-146a-5p, miR-132-3p and miR-155-5p in whole blood (**Figure 4A**). The expression results of PNP patients, with painful and painless PNP, and inflammatory and non-inflammatory PNP are displayed as fold change in comparison to UH in **Table 5**. Comparisons between PNP patients and controls, as well as between painful and painless PNP subgroups, and inflammatory and non-inflammatory did not show any differences in the expression of these components.

**Table 5:** Fold change of the gene expression of pro- and anti-inflammatory markers in whole blood from patients with PNP, in comparison to UH.

Item	PNP (Entire cohort)	Painful PNP	Painless PNP	Inflammatory PNP	Non-inflammatory PNP	UH
N	27	13	14	9	18	5
Mean ( ± SD)						
**TRPV1**	1.04 ( ± 0.24)	1.08 ( ± 0.11)	1.09 ( ± 0.14)	1.08 ( ± 0.10)	1.085 ( ± 0.13)	1.00 ( ± 0.17)
**TLR4**	1.02 ( ± 0.34)	1.06 ( ± 0.41)	0.97 ( ± 0.26)	1.05 ( ± 0.56)	1.00 ( ± 0.24)	1.00 ( ± 0.58)
**SIRT1**	0.91 ( ± 0.13)	0.92 ( ± 0.13)	0.90 ( ± 0.16)	0.86 ( ± 0.10)	0.93 ( ± 0.15)	1.00 ( ± 0.09)
**TNFα**	1.14 ( ± 0.35)	1.28 ( ± 0.25)	1.20 ( ± 0.43)	1.06 ( ± 0.23)	1.30 ( ± 0.37)	1.00 ( ± 0.36)
**IL-1β**	1.08 ( ± 0.28)	0.98 ( ± 0.32)	1.15 ( ± 0.29)	0.83 ( ± 0.14)	1.20 ( ± 0.30)	1.00 ( ± 0.45)
**IL-6**	0.79 ( ± 0.37)	0.62 ( ± 0.30)	0.78 ( ± 0.34)	0.68 ( ± 0.48)	0.72 ( ± 0.28)	1.00 ( ± 0.50)
**IL-8**	1.35 ( ± 0.99)	1.03 (0.50)	1.76 ( ± 0.43)	0.88 ( ± 0.45)	1.67 ( ± 1.29)	1.00 ( ± 0.49)
**CCL2**	0.80 ( ± 0.64)	0.69 ( ± 0.29)	1.24 ( ± 0.89)	0.60 ( ± 0.29)	1.17 ( ± 0.80)	1.00 ( ± 0.36)
**IL-10**	0.88 ( ± 0.48)	0.94 ( ± 0.49)	0.92 ( ± 0.56)	0.84 ( ± 0.53)	0.97 ( ± 0.52)	1.00 ( ± 0.33)
**miR146**	1.02 ( ± 0.31)	0.94 ( ± 0.29)	1.02 ( ± 0.30)	1.07 ( ± 0.37)	0.96 ( ± 0.27)	1.00 ( ± 0.27)
**miR132**	1.00 ( ± 0.33)	0.84 ( ± 0.21)	0.93 ( ± 0.38)	0.87 ( ± 0.27)	0.89 ( ± 0.33)	1.00 ( ± 0.29)
**miR155**	1.19 ( ± 0.57)	0.82 ( ± 0.21)	1.08 ( ± 0.42)	0.88 ( ± 0.22)	0.98 ( ± 0.40)	1.00 (0.27)

TRPV1, transient receptor potential cation channel subfamily V member 1; TLR4, toll-like receptor 4; SIRT1, sirtuin 1; TNFα, tumour necrosis factor α; IL, interleukin; CCL2, CC-chemokine ligand 2; TBP, TATA-Box binding protein; miR, microRNA, rRNA, ribosomalRNA.

Interestingly, PNP patients were next grouped into patients with and without systemic inflammation based on increased CRP levels in the serum (cut-off 0.5 mg/dl). Patients with systemic inflammation had a higher expression of IL-10 in comparison to those with normal CRP levels (≤0.5 mg/dl) (p < 0.001) (**Figure 4B**) and a positive correlation was found between CRP levels and IL-10 expression (p < 0.01) (**Figure 4E**). Furthermore, PNP patients with high CSF total protein (> 50 mg/dl) also presented lower expression of TNFα (**Figure 4C**) and higher expression of miR-155 (**Figure 4D**) than those with normal levels (≤ 50 mg/dl). Moreover, a multivariate correlation analysis (**Figure 4E**) showed that CCL2 correlated positively with the duration of the disease (p < 0.001) while negatively with several neuropathy scores such as sural nerve SNAP amplitudes (p < 0.01) or GCPS, indicating a relation between inflammation and axonal degeneration.

### Cytokine protein levels in sera

**Figure 5:**
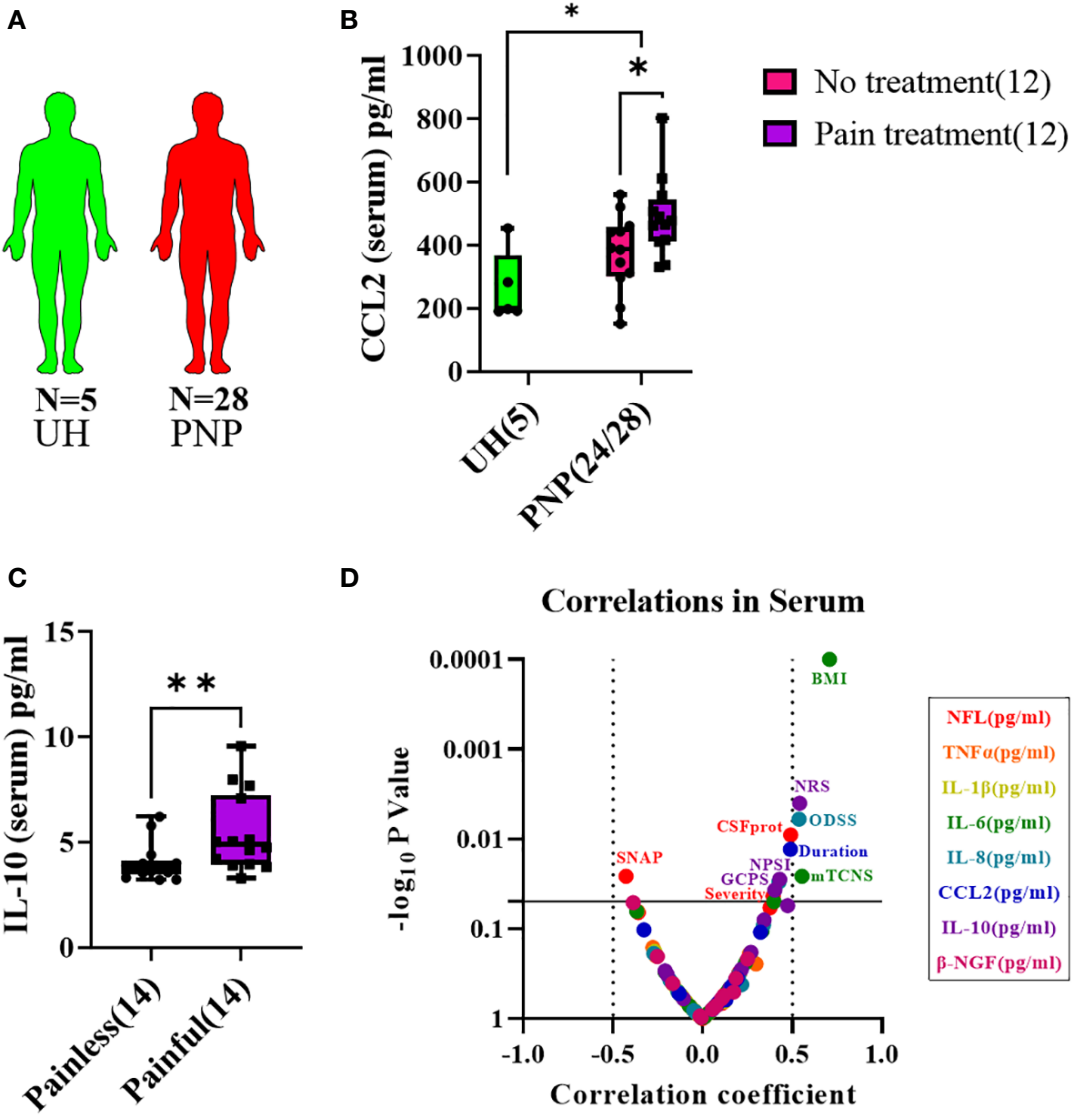
Cytokine levels in serum from patients with PNP. **(A)** Visual representation of the analyzed 5 UH (light green) and 28 PNP patients (red). **(B)** CCL2 levels in pg/ml in serum from UH and PNP patients, further divided between those without (pink) and with pain treatment (purple). **(C)** IL-10 levels in pg/ml in serum from PNP patients with painless (pink) and painful (purple) neuropathies. **(D)** Volcano plot of the Spearman multivariate correlations between each studied target and the neuropathy scores. *, p < 0.05; **, p < 0.01. All graphical images were incorporated from Smart Servier Medical Art, https://smart.servier.com/, under the Creative common Attribution 3.0 Unported Licence.

As indicators of systemic inflammation, we analyzed the levels of the pro-inflammatory cytokines TNFα, IL-1β, IL-6 and IL-8, the chemokine CCL2, and the anti-inflammatory cytokine IL-10 in sera from twenty-eight patients with PNP and five UH (**Figure 5A**). Furthermore, we measured the levels of nerve growth factor beta (NGF-β) in sera, since it is involved in nociceptive processing and a target of novel analgesics (40). The CSF from the same cohort was available and we measured the levels of the pro-inflammatory cytokines IL-6 and IL-8, and the chemokines CCL2 and fractalkine (Cx3CL1). TNFα, IL-1β and IL-10 were not detected in these samples (data not shown). All results are detailed in **Table 6**.

**Table 6:** Cytokine levels in serum and CSF from patients with PNP and UH.

Item	PNP (Entire cohort)	Painful PNP	Painless PNP	Inflammatory PNP	Non-inflammatory PNP	UH
N	28	14	14	9	19	5
Median findings in serum (range)						
**TNFα pg/ml**	9.71 (6.33-86.30)	9.87 (8.58-13.60)	9.39 (6.33-86.30)	9.77 (8.64-86.30)	9.64 (6.33-20.70)	6.78 (6.04-23.20)
**IL-1β pg/ml**	0.27 (0.04-0.61)	0.29 (0.04-0.50)	0.23 (0.12-0.61)	0.31 (0.09-0.58)	0.27 (0.04-0.61)	0.19 (0.05-0.54)
**IL-6 pg/ml**	2.94 (1.10-10.09)	3.05 (1.10-10.09	2.90 (1.27-5.87)	2.37 (1.10-10.09)	2.94 (1.12-9.36)	1.36 (0.51-3.48)
**IL-8 pg/ml**	12.60 (5.48-62.40)	14.80 (8.12-41.20)	12.50 (5.48-62.40)	12.60 (8.12-22.0)	13.00 (5.48-62.40)	9.380 (6.72-13.70)
**CCL2 pg/ml**	442.0* (151.0-802.0)	464.5 (201.0-802.0)	411.0 (151.0-560.0)	417.0 (201.0-611.0)	443.0 (151.0-802.0)	198.0 (190.0-454.0)
**IL-10 pg/ml**	4.00 (3.22-9.57)	4.89^##^ (3.29-9.57)	3.79 (3.22-6.22)	4.40 (3.22-7.69)	3.99 (3.23-9.57)	3.06 (2.63-12.50)
**NGF-β pg/ml**	4.28 (2.17-9.49)	4.58 (2.92-8.32)	3.89 (2.17-9.49)	3.53 (2.39-5.03)	4.50 (2.17-9.49)	3.34 (2.66-5.00)
Median findings in CSF (range)						
**IL-6 pg/ml**	3.32 (1.23-27.60)	3.79 (1.66-11.80)	2.81 (1.23-27.60)	5.19 (1.82-27.60)	3.21 (1.23-11.90)	5.37 (1.40-23.00)
**IL-8 pg/ml**	42.25 (14.70-180.0)	40.35 (19.40-180.0)	45.45 (14.70-124.0)	44.60 (19.40-180.0)	39.40 (14.70-124.0)	32.20 (21.80-117.0)
**CCL2 pg/ml**	415.5 (169.0-937.0)	359.0 (260.0-937.0)	422.5 (169.0-829.0)	411.0 (260.0-937.0)	414.0 (169.0-767.0)	271.0 (222.0-672.0)
**CX3CL1 pg/ml**	261.5 (89.70-500.0)	218.0 (104.0-500.0)	282.0 (89.7-444.0)	253.0 (104.0-397.0)	272.0 (89.70-500.0)	223.0 (138.0-247.0)

TNFα, Tumor necrosis factor alpha; IL, Interleukin; CCL2, CC-chemokine ligand 2, NGF-β, nerve growth factor beta; Cx3CL1, fractalkine or C-X3-C motif chemokine 1. Kruskal-Wallis or T-test was performed between PNP and UH (*), painful and painless PNP (#) and inflammatory and non-inflammatory PNP.

*, p < 0.05; ##, p < 0.01.

CCL2 was present in higher levels in serum in PNP in comparison to UH, while no differences were discovered for the other analyzed markers. CCL2 was specifically upregulated in PNP patients under analgesic treatment (p < 0.05, **Figure 5B**). No differences were discovered between the PNP subgroups inflammatory and non-inflammatory. On the other hand, patients with a painful PNP had higher levels of IL-10 in sera than those with no pain (p < 0.01, **Figure 5C**). A multivariate analysis showed that the levels of IL-10 positively correlated with the pain scores in NRS (p < 0.01), NPSI (p < 0.05) and GCPS (p < 0.05) (**Figure 5D**).

### Analysis of the relation of proteins between sera and CSF in PNP patients

The proteins present in CSF, such as NFL or inflammatory components, may be intrathecally produced or have infiltrated from the blood vessels due to a permeabilization of the B-CSF barrier. In order to study the origin of NFL and cytokines in CSF, we compared the levels of albumin between CSF and serum (albumin ratio in CSF/in serum: Q_Alb_) and divided the patients in subgroups according to the B-CSF barrier integrity: Normal B-CSF barrier integrity (Q_Alb_ < 0.007) and mild (0.007 < Q_Alb_ < 0.01), moderate (0.01 < Q_Alb_ < 0.02) and severe (Q_Alb_ > 0.02) B-CSF barrier dysfunction (**Figure S1A**) (41). Due to a low number of patients with severe B-CSF barrier dysfunction (2), moderate and severe groups were analyzed together. A moderate/severe B-CSF barrier dysfunction was present more often in inflammatory neuropathies than normal or mild dysfunction (**Figure S1B**). Furthermore, the albumin ratio (Q_Alb_) correlated with the severity of the clinical phenotype (**Figure S1C**). As expected, the levels of total protein in CSF increased as the B-CSF barrier dysfunction became more severe (**Figure S1D**), thus indicating a higher permeabilization or a relation between an intrathecal production and a B-CSF barrier dysfunction.

**Figure 6:**
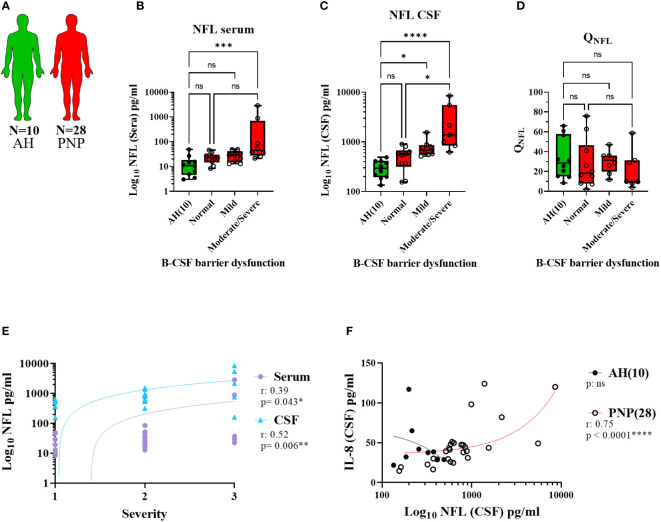
Levels of NFL between CSF and serum from patients with PNP. **(A)** Visual representation of the analyzed 5 AH (green) and 28 PNP patients (red). Levels of NFL in serum **(B)** and CSF **(C)** in patients with normal B-CSF barrier function, or mild, moderate and severe B-CSF barrier dysfunction. **(D)**. NFL ratio between CSF and serum (Q_NFL_) in AH and the subgroups of PNP patients according to their B-CSF barrier dysfunction. **(E)** Correlation between the levels of NFL in serum (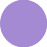) and CSF (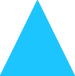) and the subjective severity. **(F)** Correlations between the levels of IL-8 and NFL in CSF in pg/ml. ns, not significant; *, p < 0.05; **, p < 0.01; ***, p < 0.001; ****, p < 0.0001. All graphical images were incorporated from Smart Servier Medical Art, https://smart.servier.com/, under the Creative common Attribution 3.0 Unported Licence.

NFL measurements in serum and CSF in the different subgroups of twenty-eight PNP patients and ten AH (**Figure 6A**) showed higher levels in those patients with moderate/severe B-CSF barrier dysfunction in comparison to those with a normal B-CSF barrier integrity and to AH (**Figures 6B, C**). This resulted in a constant Q_NFL_ (NFL ratio in CSF/serum) among subgroups and between PNP and AH (**Figure 6D**). Furthermore, NFL in serum and CSF correlated positively with the severity of the clinical phenotype (**Figure 6E**), suggesting that the release of NFL might be a consequence of the severe neurodegeneration.

**Figure 7:**
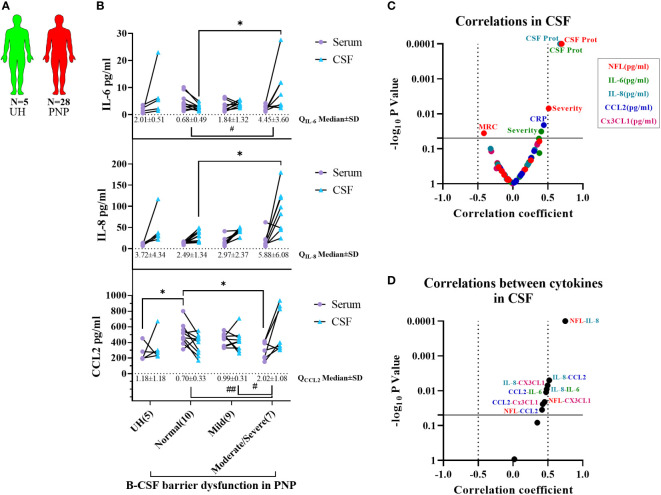
Comparison of cytokine levels between CSF and serum from patients with PNP. **(A)** Visual representation of the analyzed 5 UH (light green) and 28 PNP patients (red). **(B)** Levels of IL-6, IL-8 and CCL2 in serum (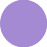) and CSF (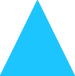) in patients with normal B-CSF barrier function, or mild, moderate and severe B-CSF barrier dysfunction. **(C)** Volcano plot of the Spearman multivariate correlations between each cytokine in CSF and the neuropathy scores **(C)**, and among each cytokine in CSF (D) */# p < 0.05; ##, p < 0.01. All graphical images were incorporated from Smart Servier Medical Art, https://smart.servier.com/, under the Creative common Attribution 3.0 Unported Licence.

Interestingly, we found a correlation between the levels of NFL and IL-8 in CSF (**Figure 6F**), thus indicating a relation between neurodegeneration and inflammation. With this discovery, we decided to study the levels of cytokines between CSF and serum, in order to determine their origin, in the different subgroups of twenty-eight PNP patients and five UH (**Figure 7A**). This analysis showed that PNP patients with a moderate/severe B-CSF barrier dysfunction present higher levels of IL-6 and IL-8 in CSF and a higher Q_IL-6_ and Q_CCL2_ (cytokine ratio in CSF/in serum), than those with a normal B-CSF barrier integrity (**Figure 7B**). A multivariate analysis showed that the levels of IL-6 in CSF correlated with the overall severity, thus indicating a relation between the production or release of IL-6 and severe PNP (**Figure 7C**). Interestingly, a second multivariate analysis showed that the levels of most cytokines analyzed in CSF correlated with each other (**Figure 7D**), therefore suggesting a common stimulus triggering their production, or their involvement in a common pathway

### Acyl-carnitine levels in serum and CSF from PNP patients

**Figure 8:**
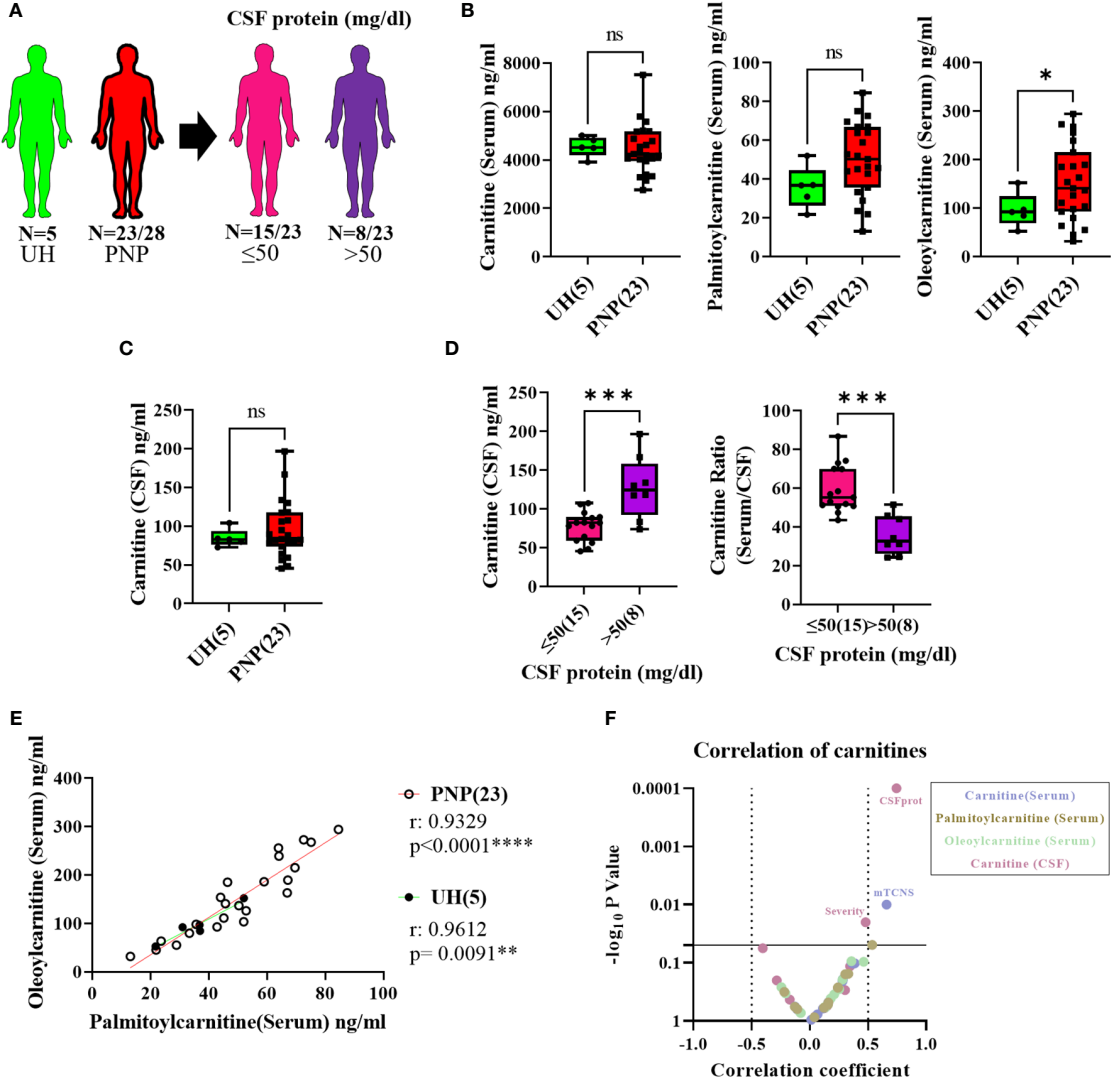
Levels of pro-inflammatory lipids in serum and CSF from patients with PNP. **(A)** Visual representation of the analyzed 5 UH (light green) and 23 out of 28 PNP patients (red), further divided into 15 PNP patients with normal (≤ 50 mg/dl) (pink) and 8 with an abnormal (> 50 mg/dl) (purple) CSF total protein. **(B)** Levels of carnitine, palmitoylcarnitine and oleoylcarnitine in ng/ml in serum from UH and PNP patients. **(C)** Levels of carnitine in ng/ml in CSF from UH and PNP patients. **(D)** Levels of carnitine in ng/ml in CSF (left) and its ratio (serum/CSF) (right) in PNP patients with normal and abnormal CSF total protein. Correlations can be found between oleoylcarnitine and palmitoylcarnitine (ng/ml) in serum from UH and PNP patients **(E)**. **(F)** Volcano plot of the Spearman multivariate correlations between each carnitine and the neuropathy scores. * p < 0.05; **, p < 0.01; ***, p < 0.001; ****, p < 0.0001. All graphical images were incorporated from Smart Servier Medical Art, https://smart.servier.com/, under the Creative common Attribution 3.0 Unported Licence.

In order to complete our study with the analysis of lipid compounds involved in pro-inflammatory pathways, we performed LC-MS to measure the levels of carnitine, palmitoylcarnitine and oleoylcarnitine in serum and CSF from twenty-three PNP patients and five UH (**Figure 8A**). While carnitine and palmitoylcarnitine in serum did not differ between PNP and UH, oleoylcarnitine was found in higher levels in patients with PNP in comparison to UH (**Figure 8B**). Carnitine was also not different in CSF between PNP patients and UH (**Figure 8C**). Palmitoylcarnitine and oleoylcarnitine were not detected in CSF samples (data not shown).

Our results also showed that PNP patients with high CSF protein (>50mg/dl) had higher levels of carnitine in CSF and a lower serum/CSF ratio than those with normal protein levels (p < 0.001) (**Figure 8D**).

Furthermore, we observed that the levels of palmitoylcarnitine and oleoylcarnitine in serum positively correlated in patients with PNP (p < 0.0001) and in UH (p < 0.001) (**Figure 8E**). Interestingly, a multivariate analysis found a positive correlation between the levels of carnitine in serum and the mTCNS (p < 0.001) as well as between the levels of carnitine in serum and the overall severity (**Figure 8F**).

## Discussion

In this study, we investigated the gene expression, protein and lipid levels of different pro-inflammatory markers in blood and CSF from patients with PNP, in comparison to a control group of patients with acute headaches of unclear etiology.

Our results showed that, contrary to our initial hypothesis of PNP being associated with enhanced systemic inflammation, PNP patients and disease controls did not present major differences in systemic inflammatory markers. Receptors of great interest in the fields of inflammation and pain, TLR4 and TRPV1, were not informative in our cohort. While only CCL2 and oleoylcarnitine were present in higher levels in sera from PNP patients than in controls, the levels of CCL2 were associated to pain treatment.

Oleoylcarnitine, as one of the long-chain acylcarnitines found upstream of the synthesis of prostaglandins and thromboxanes, has been described upregulated in patients with systemic inflammation, as well as in different neuronal tissues in models of neurodegeneration (20, 22, 24). This suggests that the levels of oleoylcarnitine indicate an inflammatory process or neurodegeneration taking place in patients with PNP. Since it did not correlate with any neuropathy scores, we postulate that oleoylcarnitine is upregulated in all patients with PNP, and it might be of interest to explore as a potential early diagnostic marker in larger groups of different types of PNP versus controls.

Secondly, we hypothesized that a systemic inflammation may correlate with the severity of the disease and the development of neuropathic pain. Our study showed that IL-10 was the only inflammatory mediator consistently upregulated at the gene and protein level in patients with severe pain, in comparison to those without pain. IL-10 is understood as an anti-inflammatory cytokine, mainly produced by anti-inflammatory macrophages, and secreted to suppress pro-inflammatory responses and maintain tissue homeostasis (42). In patients with different neuropathies, levels of IL-10 have been described downregulated in serum and CSF and negatively correlating with pain scores, therefore suggesting an increased systemic inflammation (26–30). Our study, on the other hand, showed a positive correlation between IL-10 and CRP, confirming the involvement of IL-10 in inflammatory responses, potentially in a counter-regulatory function. Our results are in accordance with previous studies where higher levels of IL-10 were reported in serum from patients with neuropathies (27, 43, 44). This suggests that the higher expression of IL-10 might act as a compensatory mechanism against the inflammation triggered by neuropathy-specific processes. However, high levels of IL-10 as well as of IL-10 expressing blood mononuclear cells have been found related to large nerve fiber sensory and motor axonal damage, as well as motor nerve demyelination (28, 45). Therefore, we cannot exclude the option that the overexpression of IL-10 might also play a direct role in the pathogenesis of nerve fiber damage

Severe neuropathy indicated by a low SNAP correlated with high gene expression of CCL2. CCL2 and its receptor CCR2 can cause hyperalgesia through the upregulation of cation channels (46–48). In addition, CCL2, also named monocyte chemoattractant protein-1 (MCP-1), is directly involved in the migration and infiltration of monocytes, memory T lymphocytes, and natural killer (NK) cells, therefore also promoting local inflammatory processes (49). High systemic levels of CCL2 might thus correlate with the development of neuropathic symptoms and could serve as a severity marker of the neuropathy. Furthermore, the high levels of CCL2 might also be due to the treatment of pain as previously mentioned. More specifically, PNP patients with the highest levels of CCL2 were those that had been treated with opioids. This could either be indicate of a molecular interaction or simply support that severe neuropathy is more likely to be painful and therefore being properly treated. Kaminski et al. provided evidence for a molecular interaction because inhibition of opioid receptors led to a downregulation of CCL2 (50). On the other hand, high levels of CCL2 can inhibit the activation of opioid receptors, thus attenuating analgesia (51, 52). This suggests that the high CCL2 levels might be a compensatory effect from the opioid treatment. Further studies need to elucidate the role of CCL2 in the development of PNP and its symptoms.

Interestingly, we found that patients with a severe B-CSF barrier dysfunction, determined by the CSF/serum albumin ratio (41), also presented higher levels of NFL in serum and CSF, as well as higher levels of IL-6 and IL-8 in CSF. NFL constitutes one of the subunits of the neurofilament that forms the cytoskeleton in neurons. Neurofilaments are especially abundant in large myelinated axons, while relatively scarce in dendrites, and their release has been described as a marker of neurodegeneration or upon neuroaxonal damage (36, 53–59). As expected, NFL levels in CSF and serum of our PNP group were increased in comparison to the controls, indicating neurodegeneration. This increase seemed to be present especially in those PNP patients with B-CSF barrier dysfunction, both in serum and CSF, while no difference was found in PNP patients with a normal B-CSF barrier and controls. The correlation we found between the albumin ratio (Q_Alb_) and the severity of the neuropathy may indicate that patients with a moderate to severe B-CSF barrier dysfunction also present more severe neuropathy and thus, more neurodegeneration, explaining the higher levels of NFL in these patients. Furthermore, while a break of the B-CSF barrier would allow the exchange of proteins between serum and CSF, the levels of NFL were consistently higher in CSF than in sera, thus indicating an intrathecal production. Since we found higher NFL levels in serum related with a decreased SNAP, and in accordance with previous studies (59–62), we believe that in diseases of the PNS, NFL in serum could come from the degeneration of peripheral axons. Since the prognosis of a neuropathy is often uncertain in an individual patient, NFL in serum could be used prospectively to monitor progress and eventually to detect unexpected accelerations of the progression. In CSF, on the other hand, differences in the levels of NFL in peripheral neuropathies have yet to be explained. In 2018, Axelsson et al. described for the first time higher NFL levels in CSF in patients with Guillain-Barré syndrome (GBS), a neuropathy of the PNS, than in controls. This increase was postulated to be due to axonal damage of nerve roots, which are surrounded by CSF in the subarachnoid space of the spinal cord (63). This idea has been restated in a more recent study in acute and chronic inflammatory polyneuropathies (57). Later in 2018, similar levels of NFL were found in CSF by Mariotto et al. in a cohort of acquired peripheral neuropathies. This study, however, discusses a possible ongoing axonal damage in both the CNS and PNS, and that the levels of NFL might be affected by a disrupted blood nerve barrier (64). Following the idea raised by Axelsson et al., we postulate that neurodegeneration takes place at the nerve roots, which constitute the very beginning of the PNS, leading to the intrathecal release of NFL. Recent studies have also found a lowered NFL CSF/Serum ratio in patients with peripheral neuropathies, therefore proposing peripheral axonal damage contributing to higher levels of NFL in serum (64, 65). Our results showed a constant CSF/serum ratio among PNP subgroups and controls, suggesting a constant increment of NFL in both tissues and therefore neurodegeneration taking place at the nerve roots as well as in peripheral nerves.

Interestingly, patients with a severe B-CSF barrier dysfunction also presented higher levels of IL-6 and IL-8 in CSF, while the levels in sera remained constant, therefore causing an increment of their CSF/serum ratio. Since 1993, high levels of IL-6 and IL-8 have been reported in CSF of patients with GBS and CIDP. This study as well as more recent ones suggest a prominent intrathecal activation of cells of the monocyte/macrophage lineage, leading to the intrathecal production of the cytokines (66–68). Following a similar line of thought, we postulate that inflammation is present at the level of the nerve roots and leads to the release of pro-inflammatory cytokines directly to the CSF. Furthermore, this inflammation may cause neuronal damage and disruption of the axonal cell membrane, inducing the releases of NFL into the CSF compartment (69). The finding of a strong correlation between the levels of IL-8 and NFL in CSF supports this assumption and leads to the question whether they are simultaneously released from the same cell type, or whether they consecutively induce each other’s release. Moreover, the levels of IL-6 in CSF correlated with the severity of neuropathy, thus indicating that the inflammation at the nerve roots might be the cause or consequence of the neurodegeneration. This would indicate that patients with more severe neuropathic symptoms would present an affection of the nerve roots and a break of the B-CSF barrier, supporting the importance of CSF analysis as a diagnostic tool for patients with PNP (70). Being an invasive procedure, lumbar puncture to obtain CSF is not without risks, and patients need to sign informed consent, however, the risk of headache, the most frequent adverse effect of lumbar puncture, can be markedly reduced by that use of an atraumatic needle (71). Other adverse effects are extremely rare when the lumbar puncture is properly performed.

Although our study includes the analysis of a large number of pro- and anti-inflammatory markers, non-biased omics-based analysis might be necessary to identify all involved markers and elucidate the specific pathways taking place in PNP. Furthermore, our results are limited by the very well characterized but low number of recruited patients and the variance in etiologies. A larger cohort might help separating the patients into males and females and into diagnostic subgroups that could lead to clearer results.

We conclude that in patients with PNP systemic inflammatory markers in blood or CSF do not differ from controls in general, but specific cytokines or lipids do. Nevertheless, we found several indications of a correlation between inflammation and the neuropathy severity and symptoms. In particular, we described a strong interaction between inflammation and neurodegeneration at the nerve roots in a specific subgroup of PNP patients with B-CSF barrier dysfunction, which highlights the importance of CSF analysis in patients with peripheral neuropathies. We believe that the diagnostic marker panel provided in our study may help improving patient stratification not only to increase diagnostic validity, but also to guide treatment decisions.

## Data availability statement

The original contributions presented in the study are included in the article/**Supplementary Material**. Further inquiries can be directed to the corresponding author.

## Ethics statement

The studies involving human participants were reviewed and approved by Würzburg Medical Faculty Ethics Committee (# 15/19 and # 242/17). The patients/participants provided their written informed consent to participate in this study.

## Author contributions

PG-F, NÜ and CS contributed to conception and design of the study. AR performed the gene expression analysis. KH and NC performed the protein analysis. KS and AN performed the lipid analysis. KH, NC, A-KR, HR, NÜ and CS contributed to recruitment of patients and collection of clinical data. A-KR and HR provided samples from healthy controls. PG-F organized the database and performed the statistical analysis. PG-F wrote the first draft of the manuscript. KS and AN wrote a section of the manuscript. All authors contributed to the article and approved the submitted version.
