# Supplementary material for: Systemic inflammatory markers in patients with polyneuropathies
Source: Front Immunol. 2023 Feb 13;14:1067714. doi: 10.3389/fimmu.2023.1067714 (PMC9969086; doi:10.3389/fimmu.2023.1067714)
Supplement: Supplementary file 1 [file DataSheet_1.docx]

**MATERIALS AND METHODS**

**Lipid analysis**

Carnitine hydrochloride, palmitoylcarnitine chloride, oleoylcarnitine hydrochloride and acetylcarnitine-d3 (D3-ACCAR) were purchased from Sigma.

Additional chromatographic parameters are detailed below:

- Analytical gradient: 0% B (0-0.5 min), 0-5% B (0.5-1.0 min) 5-95% B (1.0-2.0 min), 95% B (2.0-2.5 min), 95-100% B (2.5-2.6 min), 100% B (2.6-3.6 min), 100-0% B (3.6-3.7 min), 0% B (3.7-5.0 min)
- Column temperature: 30 °C
- Autosampler temperature: 5 °C

Detection parameters are detailed below:

- Curtain gas: 30
- CAD Gas: 6
- Gas1: 50
- Gas2: 50
- IonSpray voltage: 4500 V
- Source temperature: 550 °C

The recorded mass transitions were 162.0 → 103.1 for CAR, 400.2 → 85.1 for PC, 426.3 → 85.1 for OC and 207.1 → 85.1 for the internal standard D3-ACC.

**FIGURE LEGENDS**

**Figure S1.** **Severity of neuropathy in PNP patients with B-CSF barrier dysfunction..** A) Levels of Q_Alb_ in the control group of patients with acute headaches (AH) and in the different subgroups of PNP patients. Dotted lines show the limits between subgroups. B) Visual representation of the diagnostic subgroups to which PNP patients with different levels of B-CSF barrier dysfunction belong to. C) Correlation between Q_Alb_ and the severity of the neuropathy. D) Levels of total protein in CSF in mg/dl in AH and in PNP subgroups.

* p < 0.05; ***, p < 0.001; ****, p < 0.0001.
