# Supplementary figures and images for: Systemic inflammatory markers in patients with polyneuropathies
Source: Front Immunol. 2023 Feb 13;14:1067714. doi: 10.3389/fimmu.2023.1067714 (PMC9969086; doi:10.3389/fimmu.2023.1067714)

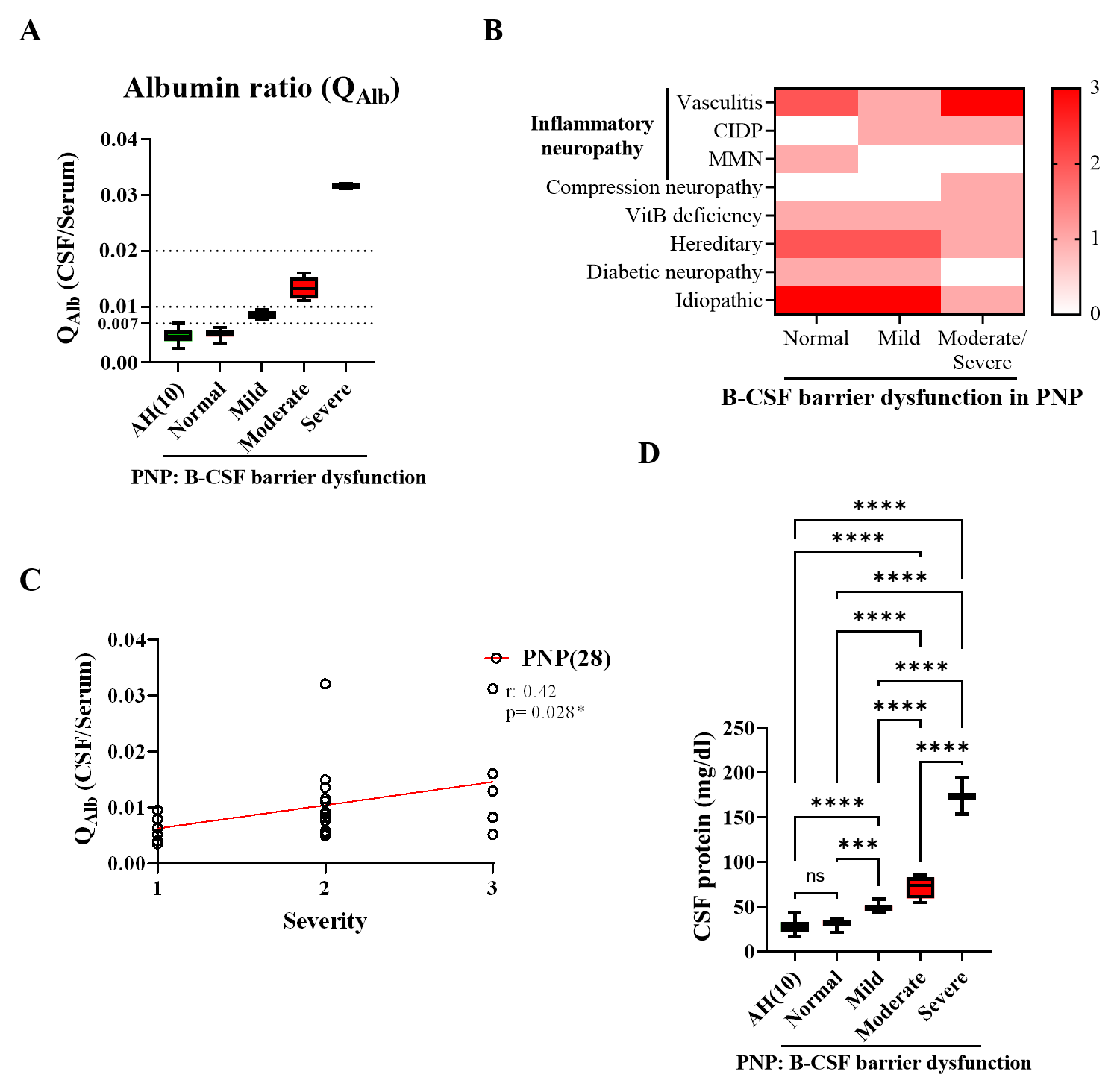

Supplement: Supplementary file 2 [file Image_1.tif]
